# Applications of Filled Single-Walled Carbon Nanotubes: Progress, Challenges, and Perspectives

**DOI:** 10.3390/nano11112863

**Published:** 2021-10-27

**Authors:** Marianna V. Kharlamova, Christian Kramberger

**Affiliations:** 1 Institute of Materials Chemistry, Vienna University of Technology, Getreidemarkt 9/BC/2, 1060 Vienna, Austria; 2 Moscow Institute of Physics and Technology, Institutskii Pereulok 9, 141700 Dolgoprudny, Russia; 3 Faculty of Physics, University of Vienna, Strudlhofgasse 4, 1090 Vienna, Austria

**Keywords:** single-walled carbon nanotube, nanoelectronics, photovoltaics, light emission, electrochemical energy storage, catalysis, sensors, spintronics, magnetic recording, biomedicine

## Abstract

Single-walled carbon nanotubes (SWCNTs), which possess electrical and thermal conductivity, mechanical strength, and flexibility, and are ultra-light weight, are an outstanding material for applications in nanoelectronics, photovoltaics, thermoelectric power generation, light emission, electrochemical energy storage, catalysis, sensors, spintronics, magnetic recording, and biomedicine. Applications of SWCNTs require nanotube samples with precisely controlled and customized electronic properties. The filling of SWCNTs is a promising approach in the fine-tuning of their electronic properties because a large variety of substances with appropriate physical and chemical properties can be introduced inside SWCNTs. The encapsulation of electron donor or acceptor substances inside SWCNTs opens the way for the Fermi-level engineering of SWCNTs for specific applications. This paper reviews the recent progress in applications of filled SWCNTs and highlights challenges that exist in the field.

## 1. Introduction

Single-walled carbon nanotubes (SWCNTs) have unique physical and chemical properties, such as electrical and thermal conductivity, mechanical strength, and flexibility, and they are ultra-light weight [1]. They are an outstanding material for applications in nanoelectronics, photovoltaics, thermoelectric power generation, light emission, electrochemical energy storage, catalysis, sensors, spintronics, magnetic recording, and biomedicine [2].

SWCNTs, also known as chirality, can be either metals or semiconductors and are solely dependent on their atomic structure. Many envisaged applications of SWCNTs necessitate nanotube samples with specific uniform electronic properties. The methods of lab-scale chirality selective synthesis and separation of SWCNTs were recently developed [3]. There are also more alternative scalable approaches to the post-synthetic chemical functionalization of SWCNTs, which allow for the controlled modification of the electronic properties of SWCNTs [4]. One example is the filling of SWCNTs. This represents a viable and flexible approach to fine-tune their electronic properties, because a large variety of substances with appropriate physical and chemical properties can be introduced inside SWCNTs [5]. The encapsulation of electron donor or acceptor substances inside SWCNTs opens the way for Fermi-level engineering of SWCNTs for specific applications.

The aim of this paper is to deliver a comprehensive review of the applications of filled SWCNTs. The structure of the review is as follows. Section 2.1 is dedicated to a description of the applications of SWCNTs in nanoelectronics. In Section 2.2, the applications of filled SWCNTs in magnetic recording devices are presented. Section 2.3 describes the applications of filled SWCNTs in nanobiotechnology. In Section 2.4, the applications of filled SWCNTs in sensors are considered. Section 2.5 highlights the applications of filled SWCNTs in the field of spintronics. In Section 2.6, the applications of filled SWCNTs in catalysis are presented. Section 2.7 considers the applications of filled SWCNTs in electrochemical storage devices. In Section 2.8, the applications of filled SWCNTs in thermoelectric power generation devices are discussed. In each section overview, reports and remarks on applications are presented.

## 2. Applications of Filled SWCNTs

### 2.1. Nanoelectronics

#### 2.1.1. Overview of Reports

State-of-the-art spectroscopic techniques and quantum-theoretical modeling have been employed frequently to study the charge transfer in filled SWCNTs. There is broad consensus that either acceptor or donor doping of SWCNTs can be achieved depending on the choice of the chemical nature of the encapsulated substance.

Acceptor doping was observed for SWCNTs filled with many different substances that can be grouped as (1) molecules—organic molecules (tetracyano-p-quinodimethane (TCNQ) and tetrafluorocyano-p-quinodimethane (F_4_TCNQ) [6,7]), fullerenes (C_60_ [8,9,10,11,12,13,14,15], C_70_, C_78_, C_82_ [12,13,14]) and endohedral fullerenes (Gd@C_82_ [16,17], La@C_82_, K@C_60_, Ca@C_60_, Y@C_60_ [10,12]); (2) simple substances—non-metals (sulfur, selenium, and tellurium [18]); and (3) chemical compounds—metal oxides (chromium (VI) oxide [19]), metal halogenides (tin (II) fluoride [20], silver chloride [21,22,23], silver halogenides [24], iron halogenides [25], cobalt bromide [26], nickel halogenides [27], iron bromide, cobalt bromide, nickel bromide [28], manganese halogenides [29,30], zinc halogenides [31], terbium chloride, zinc chloride, cadmium chloride [32], thulium chloride [33], erbium chloride [34], praseodymium chloride [35], terbium chloride, thulium chloride, praseodymium chloride [36], copper halogenides [37], copper iodide [38,39], copper chloride [40], cadmium halogenides [41], terbium halogenides [42], and potassium iodide [43,44,45]) and metal chalcogenides (gallium selenide [33,46] and gallium telluride [47]).

The list of substances that were confirmed to cause donor doping in filled SWCNTs is comparatively shorter and can be grouped into (1) molecules—organic molecules (tetrakis(dimethylamino)ethylene (TDAE) and tetrathiafulvalene (TTF) [6,7]), organometallic molecules (cobaltocene, Co(C_5_H_4_C_2_H_5_)_2_ [48,49,50], ferrocene [49,50,51,52,53,54,55,56], nickelocene [57,58,59], cerocene [60,61], and metallocenes M(C_5_H_5_)_2_, whereM = V, Cr, Mn, Ni [49]; and (2) simple substances—metals (silver [19,33,62,63,64,65,66], copper [62,67], europium [68,69], lithium, potassium [70], titanium, zinc, cobalt, nickel, iron, molybdenum, gadolinium, and copper [69,71,72,73,74,75,76,77]).

Filling of SWCNTs is a viable approach to air-stable *p-n* junctions that are required as building blocks for high-performance electronic devices and circuits. The creation of *p-n* junctions relies on the notion of partial or piecewise filling of SWCNTs with alternating electron donors and acceptors. Demonstrated examples range from partially iron-filled SWCNTs obtained by the solution method [78] to the piecewise co-filling of SWCNTs with cesium (electron donor) and iodine or C_60_ (electron acceptors) by a controlled plasma irradiation process [79].

The electrical resistivity thermal conductivity and thermopower of unfilled SWCNTs and C_60_-filled SWCNTs were measured and compared in the range from 1.5 to 300 K [80]. It was concluded that the interior linear arrangements of C_60_ chains that form an additional parallel conductive path for charge carriers act as additional sites for phonon scattering and prevent other gas molecules from absorbing at interior sites of SWCNTs. Further studies measured the transport properties of C_60_-filled SWCNT (peapods) [81,82,83,84,85]. The measurements of transport properties are complemented by calculations [15].

Authors of Reference [86] built and tested transistors with C_60_-peapods as active channels at various temperatures ranging from room temperature down to T= 1.8 K. The I/V characteristics as function of the applied gate voltage revealed single electron transistor properties in C_60_ peapods. The gate-dependent conductance was enhanced at negative gate voltages and at positive gate voltages, there was a suppressed conductance with an oscillating modulation. The modulation in the conductance correlates to the modulated density of states arising from the densely packed linear chains of C_60_ inside the peapods.

The comparison of fullerene-filled SWCNTs to SWCNTs filled with endohedral fullerenes is an interesting case as it allows for directly accessing the effects of an altered filler on the hosting SWCNTs. This opens the possibility to engineer the band gap very precisely and inspires the use of suitably filled SWCNTs as active channels with ambipolar characteristics [87,88,89,90].

**Figure 1:**
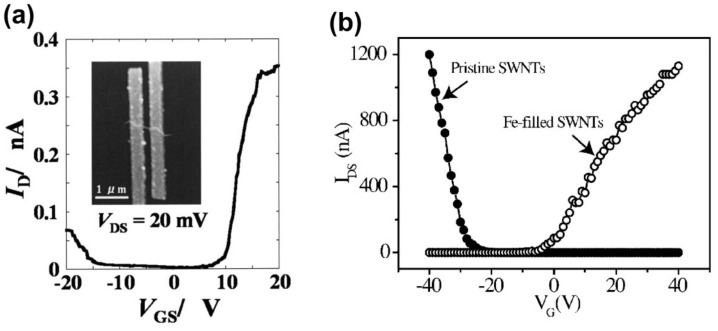
(**a**) *I_D_*–*V*_GS_ characteristics of a Gd@C_82_ metallofullerene peapods-FET (*V*_DS_=20 mV). The inset shows an atomic force micrograph of the same Gd@C_82_ peapods-FET. Reprinted from [89] with the permission of AIP Publishing. (**b**) Source-drain current (*I*_DS_) versus gate bias (*V_G_*) at a gate voltage (*V*_DS_) of 1 V for pristine SWCNTs and Fe-filled SWCNTs. Reprinted from [78] with the permission of AIP Publishing.

Field-effect transistors (FETs) with C_60_ fullerene peapods and Gd@C_82_ metallofullerene peapods as channels were used to investigate the transport properties of these two types of peapods [89]. The I/V characteristics at different gate voltages demonstrate that the C_60_-peapods-FETs exhibit *p*-type electronic characters, which is also known from FETs with semiconducting SWCNTs [91]. In stark contrast, Gd@C_82_-peapods exhibit ambipolar *p* and *n*-type electronic behavior. Gd@C_82_ peapods become conductive at finite positive and negative gate voltages. Figure 1a shows the ambipolar current versus gate voltage (*I*_D_-*V*_GS_) characteristics of a Gd@C_82_-peapods-FET at a source-drain voltage *V*_DS_ of 20 mV [89]. At positive *V*_GS_, the conductance shows a steep switching behavior with an on/off ratio on the order of 103. At negative *V*_GS_, the switching is more gradual and the achievable conductance is about four times lower. Despite the asymmetry, the results clearly prove that Gd@C_82_ peapods exhibit ambipolar FET behavior and that either *p*-type or *n*-type [92] conductance can be easily accessed by simple electrostatic gates.

In Reference [93], various fullerene nanopeapods were used as channels of field effect transistors (FET) to characterize their transport properties. C_60_ and C_78_ fullerenes, as well as Gd@C_82_ and Dy@C_82_ metallofullerenes peapods-FET show ambipolar behavior with *p* and *n*-type conductance. For the larger C_90_-peapods, all devices have shown metallic properties without any dependence on the gate voltage. All the different novel electronic properties of the peapods were explained by periodic band-gap modulations stemming from the encaged linear chains of fullerenes and metallofullerenes in nanotubes.

Authors of Reference [87] reported that filling a semiconducting SWCNT sparsely with Gd@C_82_ endohedral fullerenes effectively splits the SWCNT electronically into a series of quantum dots with lengths of about 10 nm. They mapped the spatial modulation of the band gap in the sparse semiconducting Gd@C_82_-peapods by cryogenic scanning–tunneling spectroscopy. They saw that the 0.5 eV band gap of the SWCNTs shrinks to 0.1 eV at the sites of the endohedral metallofullerenes. This modulation in the band gapis assigned to the local mechanical stress with an accompanying localized charge transfer [94] at the sites of the metallofullerene.

There is also a recent report on the temperature dependence of the conductivity type in Dy@C_82_-peapod FETs [95,96]. Chiu et al. observed a transition from *p*-type conductivity at room temperature to *n*-type conductivity at 265 K. At 215 K, there was another transition from n-type conductivity to metallic behavior.

Authors of Reference [78] created the *p-n* junction on contiguous SWCNTs by means of partial Fe encapsulation. Their transport measurements proved that the fabricated devices have an air-stable rectifying characteristic and below the threshold bias voltage there was no current down to 10^−14^ A measured. The rectifying characteristic is observed throughout the entire temperature range from 10 K to 300 K.

The source-drain current (*I*_DS_) versus gate bias (*V*_G_) measurements at a source drain voltage (*V*_DS_) of 1 V are shown in Figure 1b for pristine SWCNTs and Fe-filled SWCNTs [78]. The pristine SWCNTs exhibit the well-known *p*-type semiconducting behavior. The contiguously Fe-filled SWCNTs show the reversed *n*-type conductivity. At sparse discontinuous Fe-filling, there is also *n*-type conductivity but there are occasional cases of SWCNT devices with *p-n* junctions and rectifying characteristics.

Li et al. compared the electrical properties of FETs with channels made of ferrocene-filled SWCNTs and Fe-filled SWCNTs [97]. Their measurements conclusively show that, while Fe-filled SWCNTs exhibited high performance unipolar *n*-type characteristics, ferrocene-filled SWCNTs are indeed ambipolar semiconductors.

#### 2.1.2. Remarks on Applications

Conductivity, transparency, and mechanical robustness are very desirable in applications of SWCNT as electrodes. These properties can be improved individually by the appropriate choice of filling material to enhance the performance of transparent, stretch, and bendable devices. The challenge lies either in finding compromises or achieving multiple goals at once. Improved properties enable new designs and can help to lower the costs of production. The development in FET employing filled SWCNTs is steered towards further down-scaling the devices. While the diameter of a SWCNT is already 1 to 2 nm and cannot be further be reduced, the contact and channel length are not yet at the physical limit and can still be reduced. The filling of SWCNT can lead to better electric contacts, which would then allow for shorter contact lengths. The filling can also improve the mechanical stiffness, or, e.g., it can induce stronger magnetic moments of filled SWCNTs. These can be utilized to shorten limits in the production process on the reproducible channel lengths in integrated circuits. Practical applications of improved FET based on filled SWCNTs are also required to have high performance at and above room temperature.

### 2.2. Magnetic Recording

#### 2.2.1. Overview of Reports

Filled SWCNTs can also contain individual nanomagnets. The magnetic properties of such composites are qualitatively different than those of the same macroscopic magnetic materials in bulk. The SWCNTs also bring their own tunable properties (high electrical mobility, tunable band gap, flexibility and transparency, and very light weight) to the composite material. The combination of properties provides the means for applications in magnet-recording devices [98,99,100,101,102,103,104].

Authors of Reference [100] prepared iron nanowires inside SWCNTs and measured the ferromagnetism in the composite at room temperature. This is unexpected considering the very small diameter of nanotubes of 1.22 nm, which would for free iron nanoparticles be well in the paramagnetic regime. The hysteresis loop clearly evidences ferromagnetism with a coercive 18 mT at 300 K. In stark contrast, the unfilled iron-free SWCNT reference sample did not show any hysteresis down to 2 K. The magnetic properties of the filled SWCNTs were re-visited after several months of exposition to air and were found to be absolutely stable. This stability is attributed to the SWCNT walls preventing ambient oxidation of the iron nanowires [100].

In Reference [102] Fe-filled SWCNTs were also reported to show a higher magnetization than pristine SWCNTs. The higher magnetization decreased with increasing temperature from 5 K to 300 K. At room temperature the Fe-filled SWCNTs were ferromagnetic, while there was superparamagnetism at low temperatures.

Briones et al. [101] combined X-ray magnetic circular dichroism (XMCD) and the superconducting quantum interference device (SQUID) to investigate the orbital and spin magnetic properties of iron inside metallic and semiconducting carbon nanotubes on the atomic and macroscopic scale, respectively. The orbital and spin magnetic moments of the encapsulated iron were found to be larger inside semiconducting SWCNTs than inside metallic SWCNTs. This leads to the conclusion that the metallicity of the SWCNTs has an effect on the magnetic polarization of the encapsulated material. Briones et al. also concluded that the magnetic properties of these one-dimensional (1D) hybrid nanostructures are dominated by delocalized magnetism [101].

Kasai et al. [105] employed first-principles calculations to investigate the effects of inserting transition metals (TMs) into SWCNTs. They found that metallic isolated (3, 3) SWCNT becomes semi-metallic upon filling with Mn, Fe, and Co, while they become semiconducting upon filling with Ni. For a bundle of (3, 3) nanotube, an interstitial Co atom in the unit cell turns the bundle into a semiconductor. With two or three interstitial Co or Ni atoms in the bundle, the bundle of (3, 3) SWCNTs become a semi-metal.

In Reference [98], SWCNTs with different diameters were used to prepare nickel clusters in the inside and their magnetic properties were investigated. The control of the cluster size allowed for the preparation to synthesize cluster sizes below the exchange length of nickel. At these sizes, single-domain magnets with high coercivity emerge. X-ray diffraction (XRD) and bulk magnetization measurements were used to measure the size of the nickel clusters and their magnetization. The smallest particles exhibit superparamagnetism at room temperature and a spin glass state at low temperature.

Authors of Reference [106] reported on the transport properties of SWCNTs filled with piecewise clusters of a few hundred cobalt atoms. They observed a strong dependence of the transport properties of the device when flipping the magnetization. They concluded that cobalt nanoparticles encapsulated in SWCNTs are ferromagnetic, but with a strongly enhanced surface magnetic anisotropy. The latter forces the magnetization to be perpendicular to the host nanotube axis.

**Figure 2:**
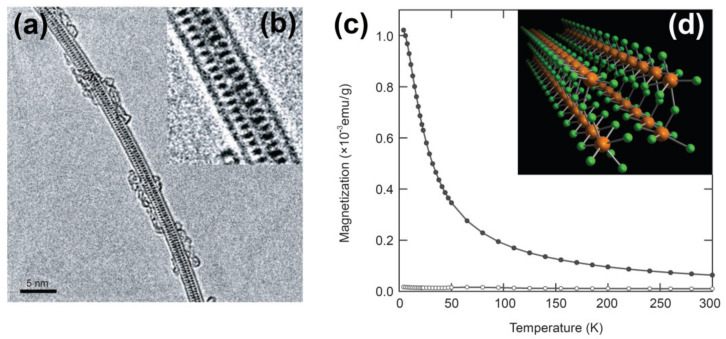
(**a**) The TEM image of the ErCl_3_-filled SWCNTs. (**b**) The high-magnification photo of the filled SWCNTs. (**c**) The magnetization of ErCl_3_-filled SWCNTs and purified empty SWCNTs as measured by SQUID magnetometry. Filled and open circles represent the magnetization of ErCl_3_-filled SWCNTs and purified empty SWCNTs. (**d**) The structural model of the encapsulated nanocrystal. Reproduced from [99]. Published by SpringerNature under a Creative Commons Attribution Non-commercial License.

The magnetic susceptibility of metal halogenide (ErCl_3_)-filled SWCNTs was measured by SQUID [99]. Figure 2a shows the transmission electron microscopy (TEM) image of the ErCl_3_-filled SWCNTs and the high-magnification photo of the filled SWCNTs is presented in Figure 2b. Figure 2c presents the magnetization of ErCl_3_-filled SWCNTs and purified empty SWCNTs as measured by SQUID magnetometry. Figure 2d shows the structural model of the encapsulated nanocrystal [99]. The magnetization of the ErCl_3_-filled SWCNTs is increased as compared to that of the pristine SWCNTs. It is accurately described by the Curie–Weiss law and both the Curie constant and Weiss temperature were determined. The Curie constant evaluates to an effective magnetic moment of 10.7μB (μB is the Bohr magnetron). This value is consistent with the 4f^11^ electronic configuration of Er^3+^ions. The Weiss temperature of −9.1K is a hallmark of a weak antiferromagnetic coupling of Er^3+^ ions [99]. The magnetization behavior of the ErCl_3_-filled SWCNTs is akin to that of bulk anhydrous ErCl_3_. This means that the magnetic coupling of Er^3+^ ions is not affected by their local atomic environment [99].

The investigations of the magnetic properties of filled SWCNTs are also complemented by calculations [73,107,108,109]. Authors of Reference [73] employed local-spin-density-functional calculations to derive the electronic and magnetic properties of SWCNTs filled with Fe nanowires. They found that in the ferromagnetic state, the magnetic moments of Fe nanowires encapsulated inside carbon nanotubes are much stronger than in bulk iron. The enhancement arises from the reduced coordination number of Fe atoms at the nanowire surface. The enhancement of the magnetic moment is more pronounced for thinner nanowires with more surface atoms. The curvature plays an additional role considering that in the thinnest Fe nanowires, the Fe atoms only interact weakly with the SWCNT walls, effectively rendering naked nanowires, while in thicker nanowires, the surface atoms interact more with the surrounding carbon, which reduces the magnetic enhancement even further.

In Reference [108], the magnetic ordering in freestanding iron nanowires and hybrids consisting of the same wires inside SWCNTs was computed from first principles. Freestanding quasi-one-dimensional *fcc* (or *hcp*) Fe-structures were found to favor ferromagnetic ordering, but when the Fe structure was encapsulated by a SWCNT, antiferromagnetic ordering was favored for some combinations of SWCNT chiralities and Fe-structures. All the isolated iron nanowires have a large spin polarization at the Fermi level, which decreases inside SWCNT due to hybridization.

Authors of Reference [109] employed ab initio methods to explore the magnetic properties of iron nanowires inside SWCNTs. They concluded that the ratio of nanowire diameter to SWCNT diameter was critical for the magnetic ordering in the combined system. If the nanowire is much thinner than the nanotube, the system is a stable ferromagnet with a large spin polarization at the Fermi level. These geometries are potentially interesting for spintronics. When the diameter of the wire approaches the one of the SWCNT, the system becomes less stable and can even undergo a transition to antiferromagnetic ordering.

#### 2.2.2. Remarks on Applications

Applications of filled SWCNT in magnetic recordings will crucially rely on the magnetic interaction at the metal–carbon interface. This specific interaction is expected to become increasingly relevant at smaller and smaller magnetic domains, and it might offer a stabilizing mechanism that would allow for tuning the paramagnetic limit for further miniaturization.

### 2.3. Nanobiotechnology

#### 2.3.1. Overview of Reports

Filled SWCNTs are also employed for bioimaging and targeted drug delivery in biomedical applications. Encapsulating chemotherapeutic drugs in SWCNTs is a way to stabilize them, improve their solubility, make their biodistrubition selective, and protect healthy tissue from their inherent toxicity [110]. SWCNTs can be thought of as stable inert transport vessels for diagnostic and therapeutic agents that prevent any aversive interaction with the surrounding biological milieu including, for instance, oxidation. The outer surface of such SWCNT vessels must be chemically functionalized with dispersing and targeting moieties [110]. For instance, specific antibodies, folic acid, or peptides can be attached to the outer surface of SWCNTs to deliver the encapsulated diagnostic and therapeutic agents to a specific tissue or even to a specific type of cells.

The foremost aim of the exterior functionalization of SWCNTs filled with diagnostic and therapeutic agents is to improve their solubility in aqueous media, as this poses the greatest challenge for biocompatibility under physiological conditions [110]. The improved aqueous solubility in the surface functionalization of SWCNTs improves their blood circulation time. At the same time, the functionalization provides a platform for further bioconjugation with specific therapeutic and/or targeting entities on the outside [110]. These provide the means for smart drug delivery, which is able target specific cell types. The cellular uptake of SWCNTs can also be tuned by the surface functionalization of SWCNTs, which can be utilized to enhance the therapeutic effect on the targeted cells [110]. All these can improve the suitability of functionalized SWCNTs for applications [111,112,113,114,115,116,117,118,119,120,121,122,123,124,125,126].

Different nanoparticles are used for biomedical applications [127]. Generally nanoparticles can penetrate biological barriers and specific nanoparticles can be used for bioimaging [128,129]. Nanoparticles can also simultaneously carry several molecular payloads and deliver a synergetic functionality [130]. The controlled release of molecular and ionic payloads [131,132] at the targeted tissue allows for conducting controlled modifications monitored by multi-parameter analysis [133].

Bioimaging of multiple concomitant processes in living cells, tissues, and whole bodies is undoubtedly a very desirable and powerful tool for diagnostics. Chemically functionalized SWCNTs filled with contrast agents hold the promise to provide such means. A pioneering bioimaging study demonstrates the monitoring of the development of abnormal processes, such as cancer development, hypoxia/hyperoxia, or necrosis [134]. A marker or contrast agent useful for bioimaging must fulfill two criteria, namely it has to support a fast and sensitive detection technique, and must also adhere to all biological requirements. The biosuitability of the contrast agent encompasses degradability and compatibility, specificity, as well as good applicability [134].

Contrast agents such as gadolinium (III) salts [135,136,137,138,139], iron oxide [140], and nitroxide radicals [141] are commonly used in magnetic resonance imaging (MRI). Pristine as well as functionalized SWCNTs filled with BiOCl/Bi_2_O_3_ [142] or radiocontrast agents such as I_2_ [143,144] and ^125^I^–^ [145,146] have been used for X-ray computer tomography. Na^125^I and ^177^LuCl_3_-filled SWCNTs [147], neutron-activated ^153^Sm sealed in SWCNT nanocapsules [148], and astanine (^211^AtCl)-filled SWCNTs [149] were put to dual-use firstly for bioimaging and secondly for radiotherapy. SmCl_3_-filled and amino-functionalized SWCNTs [150], as well as SmCl_3_-filled SWCNTs and antibody-functionalized SWCNTs [151] were engineered for targeted cancer therapy.

**Figure 3:**
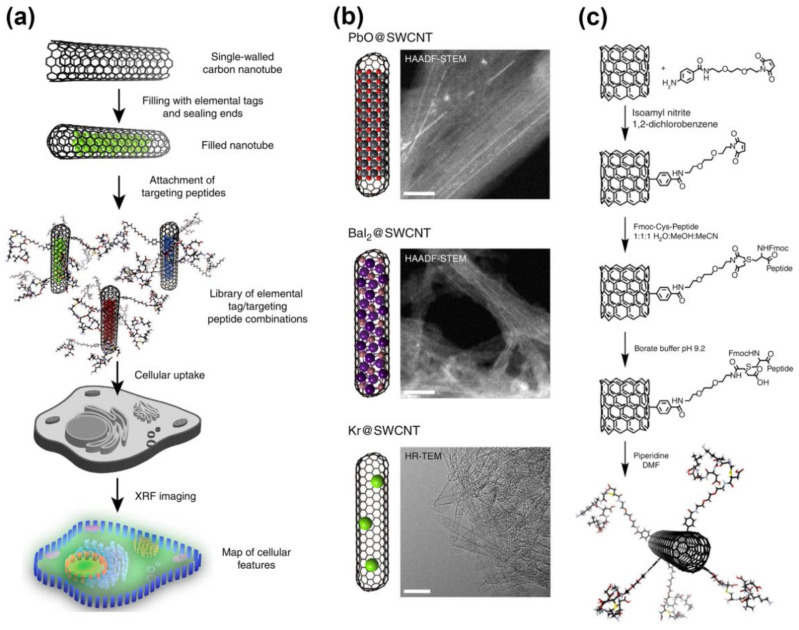
(**a**) Design of XRF-contrast carrier systems purposed to probe cellular organelles through XRF mapping. (**b**) Structural models and both HAADF-STEM and HR-TEM images of SWCNTs filled with PbO, BaI_2_, and Kr. (**c**) Scheme of functionalization of filled SWCNTs with peptides. Reproduced from [152]. Published by SpringerNature under a Creative Commons Attribution 4.0 International License.

The SWCNTs filled with PbO, BaI_2_, and Kr externally decorated with organelle-specific peptides were used as contrast agents for multiplexed X-ray fluorescence (XRF) and Raman imaging that allowed for targeting and resolving sub-cellular structures (cell membranes, nuclei, and endoplasmatic reticulum) [152]. Figure 3a shows the design of XRF-contrast carrier systems purposed to probe cellular organelles through XRF mapping. In Figure 3b, structural models and high-angle annular dark-field scanning transmission electron microscopy (HAADF-STEM) images, as well as high resolution (HR) TEM images of SWCNTs filled with PbO, BaI_2_, and Kr are presented. Figure 3c demonstrates the scheme of functionalization of filled SWCNTs with peptides [152].

The efficiency of a therapy can be dramatically increased by the use of suitably functionalized and filled nanotubes for targeted drug delivery. The key in designing a performant drug delivery system lies in water solubility, good biocompatibility, and a high blood-circulation time. Further desirable characteristics include active targeting, an efficient accumulation of therapeutics in the deceased cells and tissues, and negligible toxic effects on healthy cells or tissues. The better a properly designed smart delivery system will adhere to these requirements, the more it will also minimize any side effects of the treatment. After the therapeutic action, the entire delivery system also has to be biodegradable [153]. In recent years, efficient drug delivery systems based on SWCNTs filled with deoxyribonucleic acid (DNA) [154] and anticancer drugs such as hexamethylmelamine [155], irinotecan [156], indole [157,158], and cisplatin [159,160] were actively developed and tested. The activation of drugs upon cellular delivery could be controlled by the pH of the medium, by the temperature, or by the electric stimulation [153].

In Reference [161], molecular dynamics were used to model the system composed of carbon nanotubes and short telomeric DNA strands. The simulations suggested that these partners are able to fold into i-motif structures at slightly acidic pH conditions. The authors then continued to explore viable routes to pH-controlled drug delivery and release in such DNA–SWCNT hybrid systems. They explored two different strategies wherein doxorubicin was used as a model drug. The first strategy utilizes the pH-driven folding/unfolding of the DNA strands to realize a gate closing/opening mechanism at the ends of carbon nanotubes loaded with molecular drugs. The second strategy would suggest that the folding and unfolding DNA strands could modulate the interaction between the SWCNT and the molecular drug. In either scenario, the uptake and release of drugs by SWCNT would be switched by the pH value.

#### 2.3.2. Remarks on Applications

In bioimaging applications, filled SWCNT offers the means for improvements in the sensitivity of detection and imaging. This will not only facilitate higher spatial resolution but also the imaging of deeper laying tissues. If filled SWCNTs are employed, they can reduce cytotoxicity and improve biodegradability. They can be very beneficial to the overall biocompatibility of encapsulated contrast agents. Filled SWCNTs also hold the promise of providing a single platform that could support multimodal imaging, which would doubtlessly expand theirdiagnostic power.

Cellular drug delivery is another actively developed medical application of filled SWCNTs. The surface functionalization is further refined and new methods are developed for these applications. The development intends to boost the loading with therapeutic agents and to minimize the spurious amounts of non-loaded agents. The surface functionalization also serves the required aqueous solubility, biocompatibility, and blood-circulation time requirements. The surface functionalization also must provide the platform for specific moieties that can perform smart-targeting of specific tissues or even specifically diseased cells. Smart-targeting mitigates adverse effects for the non-targeted tissues or cells. Every use-case will require its own specifically designed targeting system, which has to be developed. The development of a biocompatible *in vitro* external trigger to release the loaded drugs is another challenge that must be overcome for the highest possible therapeutic efficiency. It is also imaginable to further expand the therapeutic options in combined therapies, wherein two or more therapeutics are co-delivered on a single platform. Similarly, a combination of imaging agents and therapeutic agents could be envisaged for combined diagnostics and per cell-level monitored treatment.

### 2.4. Sensors

#### 2.4.1. Overview of Reports

The one-dimensional electronic properties in conjunction with the very large specific surface area of SWCNTs can also be key for applications in gas sensing. The ideal gas sensor has a high selectivity, high sensitivity, fast response, fast and complete recovery, durability, and ultimately low cost. SWCNT can be employed by gas sensors if exposure to specific gases triggers a reversible change in the electric properties of bundles or isolated SWCNTs. While pristine SWCNTs are neither specific nor very sensitive towards gases, filling with a suitable substance can provide the required selectivity and sensitivity toward a certain gas.

Recently, the public has become aware that biosensors can play a critical role in managing health in populations. Fast diagnostics are essential in monitoring and managing the dynamics of pandemic outbreaks. In this context, new types of biosensors based on advanced nanomaterials can be mass-produced for readily available point-of-care diagnostics as compared to traditional centralized systems such as ELISA. Nanomaterials have unique structural [162,163], optical [164], magnetic [165,166], and electrical properties, which can be utilized to improve the sensitivity of analysis and deliver fast results, and also do not require specialized personnel.

There are biosensors based on pristine and functionalized SWCNTs for different biomolecules [167,168,169]. In Reference [170], double-walled carbon nanotubes (DWCNTs) filled with zinc iodide were employed as sensors for formaldehyde. This study proved that the sensitivity and selectivity of the sensors can be tunedfor better performance by filling the DWCNT. The filling proved to outperform the surface functionalization of nanotubes.

In References [171,172,173,174,175,176,177,178], gas sensors based on the pristine and functionalized SWCNTs were reported. Authors of Reference [171] reported on the changes in the electrical properties of SWCNTs upon the adsorption of ammonia gas at different temperatures. The SWCNTs exhibit a very strong sensitivity to NH_3_ gas. They show a detectable response for NH_3_ in concentrations as low as 5 ppm. The response scales linearly with the concentration. There are no signs of saturation in the linear response regime up to a concentration of ~40 ppm. At higher concentrations, the response becomes notably sublinear but continues to increase with increases in concentration levels.

Authors of Reference [172] tested gas sensors based on SWCNT devices for ammonia (NH_3_) in a constant flow environment. The flow was kept at 500 sccm nitrogen (N_2_) with or without a controlled concentration of 5 ppm NH_3_ for recovery and exposure. The reference value of resistance of the SWCNT-based devices was measured after annealing them and prior to any exposure to the NH_3_. After 10 min of exposure at room temperature, the resistance increased by 8%. With this setup, they could test which combinations of gas flow, sensing temperature, recovery temperature, exposure, and recovery times are appropriate for the reliable operation of a given SWCNT device. In particular, this approach allows to test sensing devices against specifications.

Fu et al. [173] reported on a scenario in which the transport properties of SWCNTs would react extremely sensitively to the electronic perturbations from environmental gases. They considered the case where already 1 ppm of CO can interact with a sidewall functionality (COOH) and trigger a change in a SWCNT resistor that can be detected at room temperature. It was suggested that a smart all-CNT electronic nose composed of two resistors (or one resistor plus one transistor) would be able to differentiate CO and other oxidative gases (for example, NO and NO_2_).

Large arrays of low-noise electrical nanotube sensors for detecting gas molecules were fabricated with 100% yield [175]. High sensitivity and selectivity of the SWCNT resistors was enabled by polymer functionalization. Polyethyleneimine coating enabled *n*-type nanotube devices to detect NO_2_ at less than 1 ppb (parts-per-billion). At the same time, it rendered them completely insensitive to NH_3_. Nafion (a polymeric perfluorinated sulfonic acid-ionomer) coating has identical complementary effects. It blocks NO_2_ and allows for selective sensing of NH_3_. A multiplexed nanotube sensor array was used to detect both species in a mixture of these molecules at once.

Authors of Reference [179] demonstrated the viability of the concept of a SWCNT-based specific gas sensor for NO_2_, which is a relevant and highly toxic air pollutant. Metallicity-sorted semiconducting and metallic SWCNTs were filled with nickel (II) acetylacetonate molecules. Both batches were compared to nickel clusters that were obtained by annealing the filled SWCNTs at 500°C. Photoemission spectroscopy was employed to show that the sensing capability of either type of SWCNTs depended on the chemical state of nickel and its local bonding environment.

**Figure 4:**
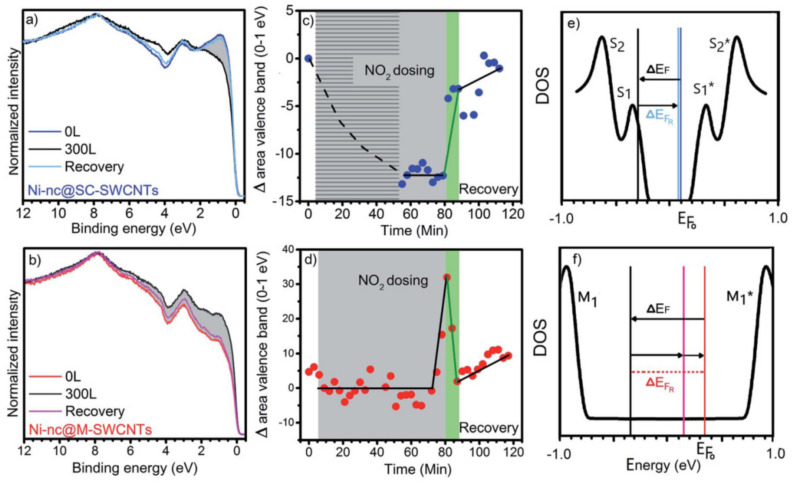
(**a**,**b**) Valence band photoemission spectra of the Ni nanocluster−filled semiconducting (**a**) and metallic (**b**) SWCNTs. In the figure, 0 L, 300 L, and recovery refer to the spectra before exposure, after exposure to 300 L of NO_2_, and after complete desorption. (**c**,**d**) Integrated area of the valence band spectra up to 1 eV as a function of the dosing and recovery time for the semiconducting (**c**) and metallic (**d**) Ni cluster−filled SWCNTs. (**e**,**f**) Sketches of the Fermi-level shifts in the density of states for Ni nanocluster-filled semiconducting (**e**) and metallic (**f**) SWCNTs. Reproduced from [179]. Published by the Royal Society of Chemistry under a Creative Commons Attribution-Non-commercial 3.0 Unported Licence.

Valence band photoemission spectra of semiconducting and metallic SWCNTs filled with Ni-clusters before exposure, after exposure to 300 L NO_2_, and after complete recovery are shown in Figure 4a,b. Figure 4c,d plot the integrated area of the valence band spectra up to 1 eV as a function of the exposure and recovery time for either type of SWCNTs. The gray shaded areas in Figure 4c,d represent 80 min of continuous exposure to NO_2_ with a total load of 300 L. The exposure is followed by 30 min of recovery. The light green stripes mark the first 3 min of the recovery period, after which the recovery is mostly completed. Figure 4e,f is sketches of the Fermi-level shifts in the density of states for semiconducting and metallic SWCNTs filled with Ni clusters, respectively [179]. It can be seen that the SWCNT gas sensor is capable of recovery after exposure at ambient temperature. The interaction between the nanotubes and the sensing target was fine-tuned by filling the SWCNTs, thus achieving gas desorption at ambient temperature.

#### 2.4.2. Remarks on Applications

Gas sensing applications of filled SWCNTs crucially depend on the issues of combined sensitivity and selectivity, quick and complete recovery, as well as lower fabrication costs. If empty SWCNTs without filling are used for gas sensing, the selectivity and durable performance cannot be achieved. A major challenge lies in the fact that the stochastic presence of surface defects and other impurities leads to an uncontrollable pattern of highly reactive sites. If an oxidizing molecule binds to one of the highly reactive sites, it interferes with the operation principle of response and recovery via chemisorption and desorption. For practical sensing applications of filled SWCNTs as gas sensors, absorption and desorption (i.e., sensing and recovery) must work at ambient conditions and many different devices have to be integrated for the miniaturization of the complete electronic noses.

### 2.5. Spintronics

#### 2.5.1. Overview of Reports

If ferromagnetic electrodes are used, SWCNTs or SWCNT networks can show spin-dependent transport [180,181,182]. Two-terminal magneto-transport measurements on SWCNT devices, in which one or two of the terminals were ferromagnetic, were reported [180]. The ferromagnetic semiconductor ((Ga and Mn)As) as well as ferromagnetic metal (Fe) electrodes have been tested. All kinds of devices exhibit a strongly hysteretic magnetoresistance below 30 K.

Non-local four-terminal magneto-transport measurements on SWCNT were reported shortly after [181]. The modeling of the device revealed that the observed magnetoresistance is consistent with a spin polarization at the contact of approximately 25%. The model also predicted that magnetoresistance changes observed earlier in two-terminal geometry are only in part because of spin accumulation.

Authors of Reference [182] reported on gate-able devices consisting of carbon nanotubes bridging ferromagnetic leads. They demonstrate a pronounced gate-field-controlled magnetoresistance. They modeled the device and showed that the sign and magnitude of the magnetoresistance are defined by the geometry of the device.

Endohedral metallofullerene molecules, for instance, Dy_n_Sc_3__-n_N@C_80_ (*n* = 1, 2), represent single-molecule magnets (SMMs) that function as isolated magnets due to their large magnetic anisotropies and slow relaxation of magnetization. They are promising materials for applications in molecular spintronics [183]. By encapsulating SMMs inside SWCNTs, the magnetic properties of SMMs are combined with strong electronic properties of SWCNTs. SMMs form a quasi-1D arrangement inside SWCNTs while being protected by the nanotube walls from the environment. Upon encapsulation, the neighboring intermolecular dipole–dipole interactions can be reduced, which leads to enhancing SMM properties. The interaction of SMMs with SWCNTs affects their electronic and spintronics properties, such as giant magnetoresistance, which are then generated [183,184,185,186,187].

Endohedral metallofullerene DySc_2_N@C_80_ were filled inside well-purified SWCNTs [188,189] with a narrow diameter distribution of 1.4 ± 0.1 nm [184]. This encapsulation achieves a controllable and uniform enhancement of SMM properties. The stepwise hysteresis characteristics of SMM are present in bulk DySc_2_N@C_80_ as well as in DySc_2_N@C_80_ inside SWCNT. However, the latter has a much higher coercivity of 4 kOe than the former at 0.5 kOe [184]. The relaxation time of the magnetization of DySc_2_N@C_80_@SWCNT was dramatically extended to ~94 min. This much longer relaxation time and higher coactivity are facilitated by the separation and reduction of the coordination number of the SMM encapsulated in the SWCNTs. The longer and fewer pairings very effectively reduce spin scattering by quantum.

Reference [183] reported on the encapsulation of SMM [Mn_12_O_12_(O_2_CCH_3_)_16_(H_2_O)_4_] in carbon nanotubes. This peapod possessed the SMM properties of the guest molecules along the functional properties of the host nanotube.

**Figure 5:**
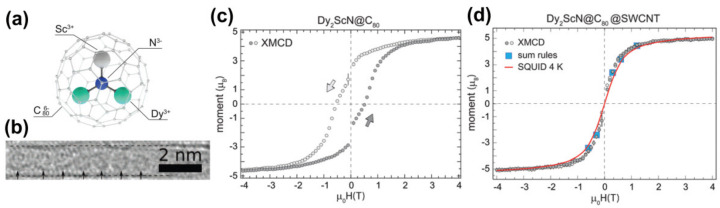
(**a**) Ball-and-stick model of Dy_2_ScN@C_80_. (**b**) HRTEM image of a chain of Dy_2_ScN@C_80_ inside a SWCNT. **(c)** Element-specific magnetization curves recorded at the Dy M_5_−edge with at 2 K of bulk Dy_2_ScN@C_80_ (**c**) and Dy_2_ScN@C_80_ encapsulated in SWCNTs (**d**). The plotted magnetization curves are the average of several independent measurements. The black error bars are the standard deviation at each external magnetic field. The scale of the magnetization curves is determined by the magnetic moments obtained by the sum-rule analysis of the XMCD data. The red magnetization curve in (**d**) was measured by a SQUID at 4 K. Reproduced from [187]. Published by the Royal Society of Chemistry under a Creative Commons Attribution-Non-commercial 3.0 UnportedLicence.

High-resolution TEM, XMCD, and ab initio calculations were combined in Reference [187] to unravel the magnetic ordering and bistability of dense linear arrangements of a SMM, namely the endohedral fullerene Dy_2_ScN@C_80_, inside SWCNTs. XMCD revealed that there is partial ordering in the orientation of the encapsulated endofullerenes, while there is no apparent order in the isotropic orientations in the bulk sample. Ab initio calculations were used to study the effect of the dense linear arrangement of the SMM on their relative orientation. The calculations showed that specific tube diameters can energetically prefer specific orientations of endohedral clusters. Element-specific magnetization curves were measured for bulk and encapsulated Dy_2_ScN@C_80_. They evidenced a lower magnetic bistability in the encapsulated Dy_2_ScN@C_80_. Figure 5a shows a labeled ball-and-stick model of Dy_2_ScN@C_80_. Figure 5b is a HRTEM micrograph of a chain of Dy_2_ScN@C_80_ inside a SWCNT. Figure 5c,d shows an element-specific magnetization curve of the bulk and encapsulated Dy_2_ScN@C_80_ [187]. The magnetization curve of bulk Dy_2_ScN@C_80_ features the characteristic low-temperature hysteresis with a typical coercive field and remanent magnetization. Very notably, the measurements performed on the encapsulated Dy_2_ScN@C_80_ sample is within entirely paramagnetic experimental errors.

Authors of Reference [190] provided a discussion on how to realize a quantum computer with endohedral fullerene-filled SWCNTs.

#### 2.5.2. Remarks on Applications

In spintronics applications, the coercivity and lifetime of magnetization in the encapsulated SMMs inside SWCNTs are the limiting factors for the achievable performance. The design goal for the maximum attainable relaxation times and thus the best possible device performance are a high yield of regularly spaced SMMs inside the SWCNTs, as well as involves ensuring the utmost purity to avoid any spurious magnetic impurities.

### 2.6. Catalysis

#### 2.6.1. Overview of Reports

The chemical inertness and thermal stability render SWCNTs with their very large internal surface area a promising support base for catalysts. They can immobilize and stabilize metal nanoparticles, and provide unique local environments for novel specialized reactions.

**Figure 6:**
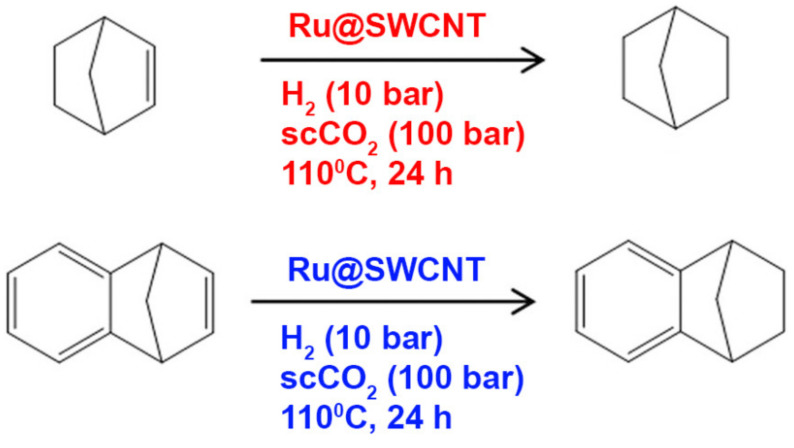
Schemes of hydrogenation reactions of norbornene and benzonorbornadiene in the presence of RuNPs encapsulated inside SWCNTs. Redrawn from [191].

In Reference [191], the catalytic properties of Ru nanoparticles (NPs) encapsulated inside SWCNTs were directly compared to external RuNPs decorating the outer surface of SWCNTs regarding hydrogenation reactions of norbornene and benzonorbornadiene (Figure 6). Supercritical CO_2_ was used to deliver the reactants to the encapsulated RuNPs. The benefit of supercritical CO_2_ was consistently even more prominent for smaller RuNPs encapsulated inside narrower SWCNTs. The SWCNTs do not only stabilize the RuNPs, but they also increase the local concentration of reactant molecules inside a local confinement. The latter effect is of comparable importance as that of metal loading or that of the size of the catalytic NPs [191]. The proposed mechanism relies on the intrinsic affinity of aromatic groups towards the negatively curved interior of SWCNTs. This selectivity boosts their local concentration and promotes the enhanced conversion of aromatic reagents in hydrogenation reactions. This effect demonstrates the ability to enhance the selectivity and efficiency of catalytic particles by hosting and engineering them in a functional matrix that actively supports the desired reactions [191].

In Reference [192], catalytic ruthenium nanoparticles were synthesized by decomposing encapsulated ruthenium carbonyl inside SWCNTs. The size of the sub-nanometer ruthenium clusters was controlled by the encapsulating SWCNTs. The SWCNTs filled with ruthenium NPs provided a unique environment for hydrogenation reactions. Different batches of SWCNTs with different mean diameters enabled the comparison of batches of differently sized NPs, which is crucial for catalytic activity. Another benefit of synthesizing the ruthenium NPs inside SWCNTs is that the predetermined NP sizes are also stabilized in the restricted interior space. This stabilization can be expected to greatly extend the lifetime of the catalytic NPs, which is exactly what was demonstrated by running multiple hydrogenation reactions over an extended period of time [192].

Authors of Reference [193] used the regular pores of alumina membranes as templates to prepare arrays of highly aligned and monodisperse graphitic carbon nanotubules. In a next step, they proceeded to prepare nanoparticles of electro-catalytic materials (i.e., Pt, Ru, and Pt/Ru) inside the carbon nanotubules. The catalyst loaded was used for gas-phase catalysis of hydrocarbons, to electrocatalyzed O_2_ reduction, and methanol oxidation.

When size-controlled Rh particles confined inside nanotubules were used for the conversion of CO and H_2_O to ethanol, there was a striking enhancement of the catalytic activity [194]. The experiments demonstrated that the production rate of ethanol (30.0 mol mol^−1^ Rh h^−1^) inside the nanotubules out competes that of free Rh particles by more than an order of magnitude. This is especially remarkable considering the accessibilities of free Rh particles and Rh nanoparticles inside the nanotubules.

#### 2.6.2. Remarks on Applications

In applications of filled SWCNTs as supports for catalytic materials or clusters, it is crucial to precisely control the size of the encapsulated particles and to effectively block the coalescence of the particles. Exerting this control on the morphology is key to improving the catalytic activity and extending the lifetime of the catalytic particles. For highly efficient and selective catalysis in filled SWCNTs, it is desirable to maximize the filling rate as well as the purity of the filled SWCNTs.

### 2.7. Electrochemical Energy Storage

#### 2.7.1. Overview of Reports

Applications in electrochemical energy storage would greatly benefit from designed hierarchical composite materials. The best-suited composites would simultaneously feature a combination of electrical conductivity, redox activity, and ion mobility. A promising approach is a composite consisting of a permeable nano-meshed conductive matrix with embedded molecular charge storage sites [195]. The internal surface area and efficient charge transfer at the interface between the two functional partners are vital for the performance of the composite.

**Figure 7:**
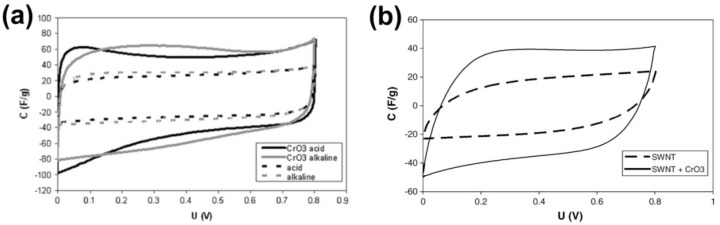
(**a**) Cylic voltammograms at 20 mV s^−1^ for CrO_3_−filled SWCNT (solid lines) and empty SWCNT (dotted lines) as electrode materials in alkaline (gray lines) and acidic (dark lines) electrolytes. (**b**) Capacitance calculated from voltammetry experiments for CrO_3_−filled SWCNT (solid lines) and empty SWCNT (dotted lines) in acidic electrolytes. Reprinted from [196] with permission from Elsevier.

In Reference [196], electrodes for supercapacitors were realized by the encapsulation of chromium oxide with high-charge capacity inside highly conductive SWCNTs. The obtained hybrid material had a very desirable combination of pseudocapacitance and conductivity for high performance electrochemical supercapacitors. Figure 7a shows cyclic voltammograms at a scan rate 20 mV s^−1^ in two types of aqueous electrolytes, i.e., 1 mol L^−1^ of H_2_SO_4_ and 6 mol L^−1^ of KOH, for electrodes made from empty SWCNT and chromium oxide-filled SWCNTs [196]. The comparison between the electrodes reveals the role of chromium oxide. The voltammograms for electrodes made of unfilled SWCNT always enclose a much smaller area, which shows that unfilled SWCNT have a more resistive character, while CrO_3_-filled SWCNTs are better capacitors. Their voltammograms are, however, irregularly shaped in the range from 0 to 0.3 V for the acidic medium and from 0 to 0.6 V in the alkaline solution. This is attributed to Faradaic redox reactions at the chromium oxide. Voltammograms at different scan rates ranging from 10 to 1000 mV s^−1^ were used to calculate the capacitance values of chromium oxide-filled SWCNT electrodes in acidic 1 mol L^−1^ of H_2_SO_4_ solution. The near-ideal shape of the voltammetry curves at the fastest scan rate of 1 V s^−1^ is shown in Figure 7b [196]. The ideal shape demonstrates that capacitors with CrO_3_-filled nanotube electrodes could easily operate at 1 V s^−1^ and still supply a capacitance of 40 F g^−1^.

Networks of SWCNTs filled with polyoxometalates (POMs) are an interesting composite system for electrochemical energy storage [195]. The nanometer-sized POMs of early transition metals (Mo, W, V, Nb, and Ta) feature a variety of oxidation stages and can undergo multiple electron redox transitions. They are promising redox components that can act the localized charge storage sites in tight contact to the highly conductive network of filled SWCNTs.

In Reference [195], the electrochemical performance of different POM-filled SWCNTs (POM@SWCNTs) in solution-phase was studied by cyclic voltammetry, namely [PW_12_O_40_]^3−^, {W_12_}, and [P_2_W_18_O_62_]^6−^, {W_18_}. The filling of the cationic SWCNTs with the anionic POMs is a self-driven process that naturally occurs in aqueous solution at room temperature. The spontaneous filling is stable and irreversible. This simplicity is key for the efficient scalable production of densely filled POM@SWCNTs hybrid materials. It was shown that the cyclic voltammograms (CVs) of {W_12_} and {W_18_} were consistent with the expected CVs of a fully functional properly interfaced POM/SWCNT hierarchical composite [195]. The CVs, after immobilizing the POM@SWCNTs on a glassy carbon electrode, showed that the encapsulated POMs maintain their electrochemical activity.

Authors of Reference [193] proposed the application of alumina membranes with carbon nanotubes in channels in fuel cells.

Several methods of opening and filling SWCNTs were developed in Reference [197] and SWCNTs filled with metal oxides were proposed as a very interesting material for electrochemical energy storage applications.

#### 2.7.2. Remarks on Applications

Electrochemical energy storage aims to maximize electrical conductance, energy density, rapid charge, and discharge, as well as cycling stability. In such applications, filled SWCNTs can provide good electrical access to the individual encapsulated redox species, as well as to the electrical conductivity across the bulk matrix. The encapsulation also serves to enhance the stability of the redox active clusters and can even enable continuous operation in reactive electrolytes that would quickly degrade free-floating unprotected redox species.

### 2.8. Thermoelectric Power Generation

#### 2.8.1. Overview of Reports

Filled SWCNTs can be organized as a hierarchical material that combines their internal mechanical strength with macroscopic low thermal conductivity and high electrical conductivity. Such characteristics are the hallmarks of materials for very efficient, flexible, and light-weight thermoelectric devices.

The electrical and thermal conductivity, as well as the thermopower, of densely C_60_-filled SWCNTs and unfilled SWCNTs were measured in the range from 1.5 to 300 K [80]. On the basis of the data, they suggested that the C_60_ chains constitute additional conductive paths for charge carriers, provide additional sites for phonon scattering, and also prevent the interior of the SWCNT from absorbing other gas molecules.

Authors of References [198,199] proposed to use the Sc_2_@C_84_ molecules-filled SWCNTs and MnTe_2_-filled SWCNTs as a nanogun.

**Figure 8:**
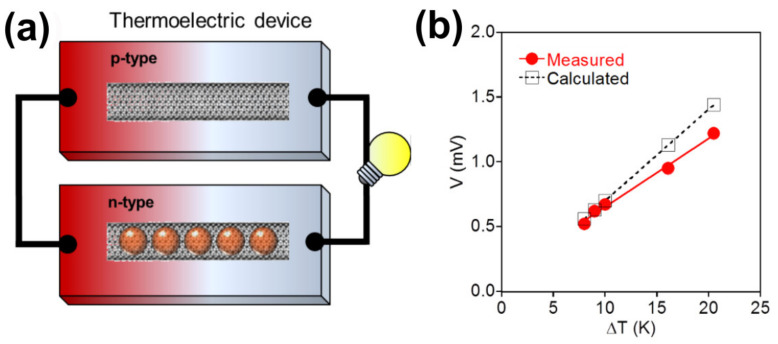
(**a**) Layout of a thermoelectric device build from empty SWCNTs and CoCp_2_-encapsulated SWCNTs. (**b**) Measured (red circle) and calculated (black square) voltage output of the thermoelectric device versus the applied temperature gradient. Reproduced from [200]. Published by SpringerNature under a Creative Commons Attribution-Non-commercial-NoDerivs 4.0 International License.

Authors of Reference [200] realized a flexible *p-n* type thermoelectric device made of two macroscopic films of SWCNTs. One consisted of naturally *p*-doped empty SWCNTs and the other film was made from *n*-doped CoCp_2_-filled SWCNTs (Figure 8a). The power generation of this kind of thermoelectric was very efficient. In fact, the device could operate close to the theoretically achievable conversion efficiency in ambient conditions without any need for protective coating.

The films of the *n*-type CoCp_2_-filled SWCNTs and empty *p*-type SWCNTs were used to fabricate a *p*-shaped thermoelectric device. The temperature difference (ΔT) across the films was controlled by heating one of the sides. In Figure 8b, the voltage is plotted against the ΔT between the two ends of the films [200]. The thermoelectric voltage at the device scales linearly with the applied ΔT. At a temperature difference of 10 K, the value amounts to 0.67 mV. The measured output is very close to the expected value (0.70 mV) based on the Seebeck coefficients of the two films (SWCNTs: ~30 μV K^−1^; CoCp_2_@SWCNT: ~−40 μV K^−1^) [200].

#### 2.8.2. Remarks on Applications

Thermoelectric applications are aimed at the highest possible conversion efficiency, which can be achieved by an increased electrical conductivity and a decreased thermal conductivity. Either conductivity can be separately engineered by choosing the right materials for filling SWCNTs for thermoelectric devices. For practical applications, the improved performance must be achievable and stable under atmospheric conditions, as well as over a wide range of temperatures. Filled SWCNTs are inert, mechanically though, flexible, and have a high thermal stability.

## 3. Conclusions

SWCNTs feature a unique combination of exceptional material properties, which are considered promising for a wide range of applications. One of the most interesting characteristics of SWCNTs is that they can either be metals or semiconductors, depending on their diameter and twist angle. While this duality attracts many potential applications, the very same applications also require an effective control of the electronic properties. The electronic properties of SWCNTs can be controlled by filling them with appropriate materials. The progress in the preparation of filled and doped SWCNTs has inspired diverse applied research. This review offers a comprehensive overview of the ongoing applied research in the exploration and development of the many different applications of filled SWCNTs.

## Data Availability

The data presented in this study are available on request from the corresponding author.

## Nomenclature

1 D	one-dimensional
CV	cyclic voltammogram
DNA	deoxyribonucleic acid
DWCNT	double-walled carbon nanotube
FET	field-effect transistor
F_4_TCNQ	tetrafluorocyano-*p*-quinodimethane
HAADF	high-angle annular dark-field
HR	high-resolution
MRI	magnetic resonance imaging
NP	nanoparticle
POM	polyoxometalate
SMM	single-molecule magnet
STEM	scanning transmission electron microscopy
SQUID	superconducting quantum interference device
SWCNT	single-walled carbon nanotube
TCNQ	tetracyano-*p*-quinodimethane
TDAE	tetrakis(dimethylamino)ethylene
TEM	transmission electron miscroscopy
TM	transition metal
TTF	tetrathiafulvalene
XMCD	X-ray magnetic circular dichroism
XRD	X-ray diffraction
XRF	X-ray fluorescence
